# First-Line Cemiplimab for Locally Advanced NSCLC: Updated Subgroup Analyses From the EMPOWER-Lung 1 and EMPOWER-Lung 3 Trials

**DOI:** 10.1016/j.jtocrr.2025.100947

**Published:** 2025-12-22

**Authors:** Ewa Kalinka, Igor Bondarenko, Miranda Gogishvili, Tamar Melkadze, Ana Baramidze, Ahmet Sezer, Tamta Makharadze, Saadettin Kilickap, Mahmut Gümüş, Konstantin Penkov, Davit Giorgadze, Mustafa Özgüroğlu, Ruben G.W. Quek, Debra A.G. McIntyre, Eric Kim, Frank Seebach, Jean-Francois Pouliot

**Affiliations:** a Polish Mother's Memorial Hospital Research Institute, Łódź, Poland; b Department of Oncology and Medical Radiology, Dnipropetrovsk Medical Academy, Dnipro, Ukraine; c High Technology Medical Centre, Tbilisi, Georgia; d Research Institute of Clinical Medicine, Todua Clinic, Tbilisi, Georgia; e Department of Medical Oncology, Medicalpark Seyhan Hospital, Adana, Turkey; f LTD High Technology Hospital Medical Center, Batumi, Georgia; g Department of Medical Oncology, Istinye University Faculty of Medicine, Istanbul, Turkey; h Department of Medical Oncology, School of Medicine, Istanbul Medeniyet University, Istanbul, Turkey; i Private Medical Institution Euromedservice, St Petersburg, Russia; j State Budgetary Healthcare Institution of Omsk Region, Omsk, Russia; k Cerrahpaşa Medical Faculty, Istanbul University-Cerrahpaşa, Istanbul, Turkey; l Regeneron Pharmaceuticals, Inc., Tarrytown, New York, USA

**Keywords:** Cemiplimab, Immunotherapy, Immune checkpoint inhibitors, Locally advanced non–small cell lung cancer, PD-L1

## Abstract

**Introduction:**

Patients with unresectable locally advanced NSCLC who are not candidates for concurrent chemoradiation represent an unmet medical need. We report long-term results for this patient subgroup from two phase III trials.

**Methods:**

We analyzed data from patients with locally advanced NSCLC in the EMPOWER-Lung 1 (NCT03088540) and EMPOWER-Lung 3 (NCT03409614) studies. In EMPOWER-Lung 1, patients were randomized 1:1 to first-line (1L) cemiplimab monotherapy or chemotherapy with more than or equal to 50% programmed death-ligand 1 expression. In EMPOWER-Lung 3, patients were randomized 2:1 to 1L cemiplimab plus chemotherapy or chemotherapy, regardless of programmed death-ligand 1 expression.

**Results:**

Patients with locally advanced NSCLC constituted 15% of the overall study populations. With cemiplimab monotherapy, the overall survival (OS) was improved versus chemotherapy (median 26.1 versus 13.9 mo; hazard ratio [HR]: 0.67, 95% confidence interval [CI]: 0.38–1.17) and progression-free survival (8.1 versus 6.2 mo; HR: 0.56, 95% CI: 0.34–0.95). With cemiplimab plus chemotherapy, the OS was improved versus chemotherapy alone (24.1 versus 13.8 mo; HR: 0.50, 95% CI: 0.27–0.95) and progression-free survival (12.5 versus 6.2 mo; HR: 0.34, 95% CI: 0.19–0.61). Treatment-emergent adverse events grade more than or equal to 3 occurred in 37.8% (cemiplimab) and 53.7% (chemotherapy) in EMPOWER-Lung 1 and in 46.7% (cemiplimab plus chemotherapy) and 25.0% (chemotherapy) in EMPOWER-Lung 3. Favorable patient-reported outcomes were observed with cemiplimab monotherapy than chemotherapy; no significant patient-reported outcomes favoring chemotherapy were observed in either study.

**Conclusions:**

This subgroup analysis supports the clinical benefit of 1L cemiplimab as monotherapy or combined with chemotherapy in patients with unresectable locally advanced NSCLC (not candidates for definitive chemoradiotherapy).

## Introduction

Stage 3 locally advanced NSCLC accounts for up to one-third of diagnoses of NSCLC and is a heterogenous disease with large differences in tumor size, local infiltration, and lymph node involvement.^1^ For patients with unresectable disease, treatment has historically been suboptimal, with 5-year survival rates ranging from 10% to 30%.^1^^,^^2^ Concurrent chemoradiation therapy (CRT) has evolved to be the preferred treatment for patients who are fit, with improved 5-year survival outcomes, though it has also been associated with a risk of grade 3 and above esophagitis.^3^

For patients who undergo concurrent CRT, consolidation immunotherapy with durvalumab is currently the standard of care,^4^^,^^5^ with increased overall survival (OS) and progression-free survival (PFS) as observed in the phase 3 PACIFIC trial.^6^^,^^7^ This trial included only those patients who completed concurrent CRT without disease progression and thus may not represent the full population intended to receive chemoradiation. More recently, the PACIFIC-2 study revealed that adding durvalumab to concurrent chemoradiation for unresectable stage 3 disease did not improve outcomes as compared with chemoradiation alone,^8^ suggesting that treatment escalation may not be the best option and that some patients may require a more conservative approach.

Many patients with unresectable stage 3 locally advanced NSCLC are not candidates for curative-intent concurrent CRT due to several factors, including contraindications to CRT, weight loss, poor performance status, significant comorbidity, or patient choice.^9^^,^^10^ These patients have historically been excluded from metastatic immunotherapy trials, and thus there are very limited data from prospective clinical studies to inform treatment.^11^, ^12^, ^13^, ^14^, ^15^ With platinum-based chemotherapy often remaining the only standard of care available, there is an unmet medical need for additional treatment options in the first-line setting for patients with unresectable locally advanced NSCLC.

Patients with stage 3B/C locally advanced NSCLC who were not candidates for surgery or concurrent chemoradiation therapy were included as part of the study population in two phase 3 clinical trials of cemiplimab, EMPOWER-Lung 1 and EMPOWER-Lung 3. EMPOWER-Lung 1 (NCT03088540) investigated cemiplimab monotherapy in patients with programmed death-ligand 1 (PD-L1) tumor expression of more than or equal to 50%,^16^^,^^17^ whereas EMPOWER-Lung 3 part 2 (NCT03409614) investigated the combination of cemiplimab with chemotherapy for patients with NSCLC regardless of PD-L1 expression level.^18^^,^^19^ Both trials demonstrated significant improvement in OS, PFS, and objective response rate (ORR) for cemiplimab compared with chemotherapy, including acceptable safety profiles. These results supported U.S. Food and Drug Administration and European Medicines Agency approvals of cemiplimab as a first-line treatment for advanced NSCLC (metastatic or locally advanced disease not suitable for definitive chemoradiation) with no *EGFR*, *ALK*, or *ROS1* genomic aberrations.^20^^,^^21^

In this subgroup analysis, we report efficacy, safety, and patient-reported outcomes (PROs) for patients with locally advanced NSCLC (approximately 15% of the total trial populations) from EMPOWER-Lung 1 and EMPOWER-Lung 3.

## Material and Methods

### Study Design and Patients

EMPOWER-Lung 1 (NCT03088540) was a multicenter, open-label, global, phase 3 randomized (1:1) controlled trial of cemiplimab monotherapy versus investigator’s choice of platinum-doublet chemotherapy in the first-line treatment of patients with advanced NSCLC whose tumors express PD-L1 in more than or equal to 50% of tumor cells (Supplementary Fig. 1).^16^ Patients were randomized 1:1 and stratified by histology (squamous versus nonsquamous) and geographic region (Europe, Asia, or the rest of the world) to either cemiplimab 350 mg administered intravenously every 3 weeks for up to 108 weeks (i.e., up to 36 treatment cycles) or 4 to 6 cycles of investigator’s choice of platinum-doublet chemotherapy.

EMPOWER-Lung 3 (NCT03409614) was a two-part, randomized (2:1), phase 3 study which compared first-line cemiplimab plus chemotherapy versus placebo plus chemotherapy in patients with advanced NSCLC and any PD-L1 expression level (Supplementary Fig. 1).^18^ Patients were randomized 2:1 and stratified by histology (squamous versus nonsquamous) and PD-L1 expression level (<1%, 1%–49%, and ≥50%) to receive either cemiplimab 350 mg or placebo every 3 weeks in combination with four cycles of investigator’s choice of chemotherapy. Patients were treated for up to 108 weeks or until disease progression or unacceptable toxicity.

Both studies included patients with squamous or nonsquamous NSCLC that was metastatic or locally advanced (not suitable for definitive concurrent CRT) without *EGFR*, *ALK*, or *ROS1* genomic aberrations. Detailed methods and eligibility criteria have been published previously.^16^^,^^18^

This subgroup analysis evaluated efficacy, safety, and PROs for the subgroup of patients with stage 3B/C locally advanced NSCLC from EMPOWER-Lung 1 and EMPOWER-Lung 3 part 2. A similar analysis was performed for patients with metastatic NSCLC.

### Outcomes and Statistical Analyses

Radiographic tumor assessments and efficacy end points including OS, PFS, ORR, and duration of response (DOR) were previously reported.^16^^,^^18^ In this analysis, efficacy end points were analyzed among patients with locally advanced NSCLC and metastatic NSCLC in each trial. Safety was assessed, with adverse events (AEs) graded according to the National Cancer Institute Common Terminology Criteria for Adverse Events version 4.03. PROs were evaluated for the locally advanced NSCLC subgroup populations for both studies. PRO methodology was published previously^22^^,^^23^ and is summarized in the Supplementary Methods. For EMPOWER-Lung 1, outcomes were assessed in the PD-L1 more than or equal to 50% population with verified PD-L1 levels. For EMPOWER-Lung 3 part 2, outcomes were assessed in the intention-to-treat population. Safety was assessed in patients who received at least one dose of assigned treatment.

For EMPOWER-Lung 1, efficacy and safety data are based on a data cutoff date of March 4, 2022; PROs are reported using a data cutoff date of March 1, 2020. For EMPOWER-Lung 3 part 2, efficacy and safety data are based on a data cutoff date of June 14, 2022; PROs are reported using a data cutoff date of June 14, 2021.

Statistical analyses for EMPOWER-Lung 1 and EMPOWER-Lung 3 part 2 have been published previously^16^^,^^18^ and are summarized in the Supplementary Methods.

### Trial Oversight

The study protocols (and all amendments) for EMPOWER-Lung 1 and EMPOWER-Lung 3 were approved by the institutional review board or independent ethics committee at each participating study site. Both studies were conducted in accordance with the principles of the Declaration of Helsinki and the International Conference on Harmonisation Good Clinical Practice guidelines. All patients provided written informed consent before participation. Details of ethical approvals were reported in primary study publications.^16^^,^^18^

## Results

### Baseline Demographic and Clinical Characteristics for Locally Advanced NSCLC Subgroups

Patients with locally advanced NSCLC comprised 15.4% (n = 87/565) of participants in EMPOWER-Lung 1 and 14.8% (n = 69/466) of participants in EMPOWER-Lung 3 part 2. Baseline demographic and clinical characteristics were generally consistent between treatment and control groups within each trial and were comparable to the overall patient population (Table 1).

**Table 1:** Patient Demographics and Baseline Characteristics in Patients With Locally Advanced NSCLC

Characteristic	EMPOWER-Lung 1 (n = 565)			EMPOWER-Lung 3 Part 2 (n = 466)		
	Cemiplimab (n = 45)	Chemotherapy (n = 42)	Total (N = 87)	Cemiplimab + Chemotherapy (n = 45)	Placebo + Chemotherapy (n = 24)	Total (N = 69)
Age, y, median (range)	63.0 (31–79)	63.5 (43–81)	63.0 (31–81)	62.0 (39–82)	63.0 (52–77)	62.0 (39–82)
≥65, n (%)	17 (37.8)	19 (45.2)	36 (41.4)	15 (33.3)	10 (41.7)	25 (36.2)
Male, n (%)	41 (91.1)	34 (81.0)	75 (86.2)	37 (82.2)	20 (83.3)	57 (82.6)
ECOG PS, n (%)						
0	16 (35.6)	13 (31.0)	29 (33.3)	10 (22.2)	3 (12.5)	13 (18.8)
1	29 (64.4)	29 (69.0)	58 (66.7)	35 (77.8)	21 (87.5)	56 (81.2)
Smoking status, n (%)						
Current	22 (48.9)	16 (38.1)	38 (43.7)	28 (62.2)	13 (54.2)	41 (59.4)
Past	23 (51.1)	26 (61.9)	49 (56.3)	11 (24.4)	8 (33.3)	19 (27.5)
Never	–	–	–	6 (13.3)	3 (12.5)	9 (13.0)
Histology, n (%)						
Squamous	27 (60.0)	28 (66.7)	55 (63.2)	23 (51.1)	13 (54.2)	36 (52.2)
Nonsquamous	18 (40.0)	14 (33.3)	32 (36.8)	22 (48.9)	11 (45.8)	33 (47.8)
PD-L1 expression level, n (%)						
<1%	–	–	–	15 (33.3)	7 (29.2)	22 (31.9)
1%–49%	–	–	–	16 (35.6)	9 (37.5)	25 (36.2)
≥50%	45 (100)	42 (100)	87 (100)	14 (31.1)	8 (33.3)	22 (31.9)

*Note:* Data cutoff date for EMPOWER-Lung 1: March 4, 2022. Data cutoff date for EMPOWER-Lung 3: June 14, 2022.

ECOG PS, Eastern Cooperative Oncology Group performance status; PD-L1, programmed death-ligand 1.

### Efficacy Outcomes for Locally Advanced NSCLC Subgroups

**Figure 1:**
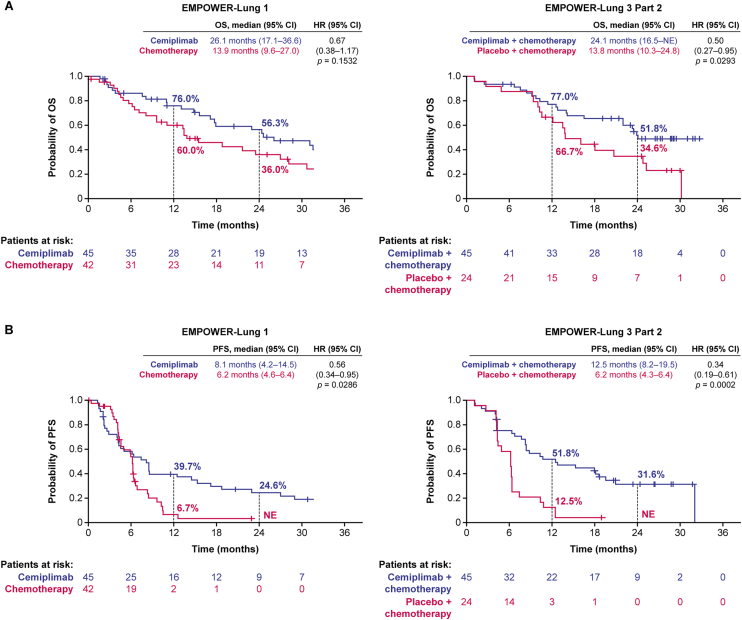
(*A*) OS and (*B*) PFS in patients with locally advanced NSCLC. Kaplan–Meier curves for OS and PFS were represented for cemiplimab versus placebo and cemiplimab + chemotherapy versus placebo + chemotherapy. Tick marks indicate censored observations, vertical lines indicate the times of landmark OS or PFS analyses, and percentages at the times of landmark PFS analysis indicate the probability of PFS or OS. Data cutoff date for EMPOWER-Lung 1: March 4, 2022. Data cutoff date for EMPOWER-Lung 3: June 14, 2022. CI, confidence interval; HR, hazard ratio; NE, not evaluable; OS, overall survival; PFS, progression-free survival.

In EMPOWER-Lung 1, at a median follow-up of 35.7 months, first-line cemiplimab monotherapy led to a median OS of 26.1 months versus 13.9 months with chemotherapy (hazard ratio [HR]: 0.67; 95% confidence interval [CI]: 0.38–1.17) for patients with locally advanced NSCLC (Fig. 1*A*); median PFS was 8.1 months versus 6.2 months, respectively (HR: 0.56; 95% CI: 0.34–0.95; Fig. 1*B*).

The ORR was 48.9% (22/45) for cemiplimab and 31.0% (13/42) for chemotherapy, with a longer median DOR for cemiplimab than for chemotherapy (median [95% CI]: 18.8 [6.4–not estimable] versus 6.2 [3.4–8.5] mo; Table 2).

**Table 2:** Summary of Tumor Responses in Patients With Locally Advanced NSCLC

Characteristic	EMPOWER-Lung 1^a^ (n = 565)		EMPOWER-Lung 3 Part 2 (n = 466)	
	Cemiplimab (n = 45)	Chemotherapy (n = 42)	Cemiplimab + Chemotherapy (n = 45)	Placebo + Chemotherapy (n = 24)
Duration of follow-up, mo, median (range)^b^	36.24 (24.4–53.7)	35.63 (24.3–53.6)	28.68 (21.0–35.9)	29.85 (22.6–35.4)
ORR, n (%)	22 (48.9)	13 (31.0)	26 (57.8)	7 (29.2)
95% CI	33.7–64.2	17.6–47.1	42.2–72.3	12.6–51.1
Odds ratio (95% CI)	2.08 (0.88–4.95)		3.32 (1.15–9.60)	
Best overall response, n (%)				
Complete response	2 (4.4)	1 (2.4)	4 (8.9)	0
Partial response	20 (44.4)	12 (28.6)	22 (48.9)	7 (29.2)
Stable disease	10 (22.2)	21 (50.0)	16 (35.6)	16 (66.7)
Noncomplete response/nonprogressive disease	0	1 (2.4)	0	0
Progressive disease	10 (22.2)	1 (2.4)	1 (2.2)	0
Not evaluable	3 (6.7)	6 (14.3)	2 (4.4)	1 (4.2)
Kaplan–Meier estimated DOR, mo, median (95% CI)	n = 22 18.8 (6.4–NE)	n = 13 6.2 (3.4–8.5)	n = 26 27.8 (13.1–27.8)	n = 7 4.2 (3.0–10.3)

*Note:* Radiographic tumor assessments and efficacy end points, including OS, PFS, ORR, and DOR, were previously reported for EMPOWER-Lung 1^16^ and EMPOWER-Lung 3 part 2.^18^

CI, confidence interval; DOR, duration of response; NE, not estimable; ORR, objective response rate; OS, overall survival; PD-L1, programmed death-ligand 1; PFS, progression-free survival.

a PD-L1 ≥50% population.

b From randomization to the data cutoff date.

In EMPOWER-Lung 3, at a median follow-up of 29.0 months, greater efficacy was observed with first-line cemiplimab plus chemotherapy versus placebo plus chemotherapy for patients with locally advanced NSCLC: median OS was 24.1 months versus 13.8 months (HR: 0.50; 95% CI: 0.27–0.95; Fig. 1*A*) and median PFS was 12.5 months versus 6.2 months (HR: 0.34; 95% CI: 0.19–0.61; Fig. 1*B*). The ORR was 57.8% (26/45) for cemiplimab in combination with chemotherapy versus 29.2% (7/24) for chemotherapy alone; there was also a longer median DOR for cemiplimab plus chemotherapy than for chemotherapy alone (median [95% CI] 27.8 [13.1–27.8] versus 4.2 [3.0–10.3] mo, respectively) (Table 2).

### Safety Outcomes for Locally Advanced NSCLC Subgroups

Among patients with locally advanced NSCLC, the median (range) duration of exposure was longer for the treatment group than for the control group in both studies: 38.9 (6.0–120.1) weeks versus 18.0 (1.7–96.4) weeks in EMPOWER-Lung 1 and 69.6 (4.7–128.1) versus 31.7 (4.9–83.9) weeks in EMPOWER-Lung 3, respectively (Table 3).

**Table 3:** Safety Summary in Patients With Locally Advanced NSCLC

Characteristic	EMPOWER-Lung 1^a^ (n = 565)		EMPOWER-Lung 3 Part 2 (n = 466)	
	Cemiplimab (n = 45)	Chemotherapy(n = 41)	Cemiplimab + Chemotherapy (n = 45)	Placebo + Chemotherapy (n = 24)
Duration of exposure, median (range), wk	38.9 (6.0–120.1)	18.0 (1.7–96.4)	69.6 (4.7–128.1)	31.7 (4.9–83.9)
TEAEs regardless of attribution, n (%)				
Any grade	41 (91.1)	40 (97.6)	45 (100)	24 (100)
Grade ≥3	17 (37.8)	22 (53.7)	21 (46.7)	6 (25.0)
Any grade leading to discontinuation	3 (6.7)	2 (4.9)	4 (8.9)	0
Any grade resulting in death	4 (8.9)	4 (9.8)	3 (6.7)	2 (8.3)
Grade ≥3 TEAEs occurring in ≥5% of patients in either arm in either trial, n (%)^a^				
Pneumonia	4 (8.9)	1 (2.4)	0	0
Anemia	1 (2.2)	5 (12.2)	2 (4.4)	2 (8.3)
Neutropenia	1 (2.2)	5 (12.2)	3 (6.7)	1 (4.2)
Any-grade TEAEs occurring in ≥1% of patients in either arm or in either trial, n (%)^a^				
Arthralgia	7 (15.6)	2 (4.9)	7 (15.6)	3 (12.5)
Back pain	7 (15.6)	4 (9.8)	3 (6.7)	1 (4.2)
Anemia	6 (13.3)	19 (46.3)	18 (40.0)	8 (33.3)
Cough	6 (13.3)	3 (7.3)	3 (6.7)	1 (4.2)
Headache	6 (13.3)	0	1 (2.2)	0
Pneumonia	6 (13.3)	2 (4.9)	2 (4.4)	0
Fatigue	5 (11.1)	4 (9.8)	6 (13.3)	3 (12.5)
Peripheral edema	5 (11.1)	0	1 (2.2)	0
Rash	5 (11.1)	2 (4.9)	2 (4.4)	1 (4.2)
Diarrhea	4 (8.9)	3 (7.3)	5 (11.1)	2 (8.3)
Decreased appetite	3 (6.7)	8 (19.5)	5 (11.1)	5 (20.8)
Decreased weight	3 (6.7)	2 (4.9)	4 (8.9)	4 (16.7)
Hemoptysis	3 (6.7)	3 (7.3)	5 (11.1)	2 (8.3)
Hyperglycemia	3 (6.7)	2 (4.9)	11 (24.4)	4 (16.7)
Hypothyroidism	3 (6.7)	0	8 (17.8)	1 (4.2)
Increased aspartate aminotransferase	3 (6.7)	2 (4.9)	7 (15.6)	2 (8.3)
Insomnia	3 (6.7)	2 (4.9)	8 (17.8)	2 (8.3)
Non-cardiac chest pain	3 (6.7)	2 (4.9)	6 (13.3)	3 (12.5)
Increased alanine aminotransferase	2 (4.4)	1 (2.4)	9 (20.0)	3 (12.5)
Peripheral sensory neuropathy	2 (4.4)	1 (2.4)	9 (20.0)	3 (12.5)
Asthenia	1 (2.2)	3 (7.3)	5 (11.1)	4 (16.7)
COVID-19	1 (2.2)	0	5 (11.1)	0
Increased blood creatinine	1 (2.2)	2 (4.9)	6 (13.3)	3 (12.5)
Increased blood lactate dehydrogenase	1 (2.2)	0	5 (11.1)	1 (4.2)
Neutropenia	1 (2.2)	13 (31.7)	9 (20.0)	1 (4.2)
Alopecia	0	10 (24.4)	18 (40.0)	12 (50.0)
Decreased platelet count	0	3 (7.3)	5 (11.1)	1 (4.2)
Increased weight	0	0	6 (13.3)	0
Nausea	0	14 (34.1)	14 (31.1)	5 (20.8)
Thrombocytopenia	0	9 (22.0)	1 (2.2)	2 (8.3)
Vomiting	0	8 (19.5)	8 (17.8)	2 (8.3)

*Note*: Grade ≥3 TEAEs were reported in ≥5% of patients and any-grade TEAEs were reported in ≥1% of patients in either arm in either trial. AEs were graded according to the National Cancer Institute Common Terminology Criteria for Adverse Events version 4.03.

Data cutoff date for EMPOWER-Lung 1: March 4, 2022. Data cutoff date for EMPOWER-Lung 3: June 14, 2022.

AE, adverse event; COVID-19, coronavirus disease 2019; TEAE, treatment-emergent adverse event.

a The events are listed in descending order of frequency in the cemiplimab arm in the EMPOWER-Lung 1 trial.

In EMPOWER-Lung 1, the most frequently occurring treatment-emergent AEs were arthralgia (15.6%) and back pain (15.6%) in the cemiplimab group and anemia (46.3%), nausea (34.1%), neutropenia (31.7%), and alopecia (24.4%) in the chemotherapy group. In EMPOWER-Lung 1, AEs of grade 3 or above occurred in 17 patients (37.8%) receiving cemiplimab and 22 patients (53.7%) receiving chemotherapy (Table 3).

In EMPOWER-Lung 3, the most frequently occurring treatment-emergent AEs were anemia (40.0%), alopecia (40.0%), nausea (31.1%), and hyperglycemia (24.4%) in the cemiplimab plus chemotherapy group, whereas anemia (33.3%), alopecia (50.0%), nausea (20.8%), and decreased appetite (20.8%) were most often reported with chemotherapy alone. In EMPOWER-Lung 3, AEs of grade 3 or above occurred in 21 patients (46.7%) receiving cemiplimab plus chemotherapy and six patients (25.0%) receiving chemotherapy (Table 3).

In EMPOWER-Lung 1, three patients in the cemiplimab arm and two in the chemotherapy arm experienced AEs leading to treatment discontinuation; in EMPOWER-Lung 3, this occurred in four patients in the cemiplimab plus chemotherapy arm and none in the chemotherapy arm.

### Patient-Reported Outcomes for Locally Advanced NSCLC Subgroups

**Figure 2:**
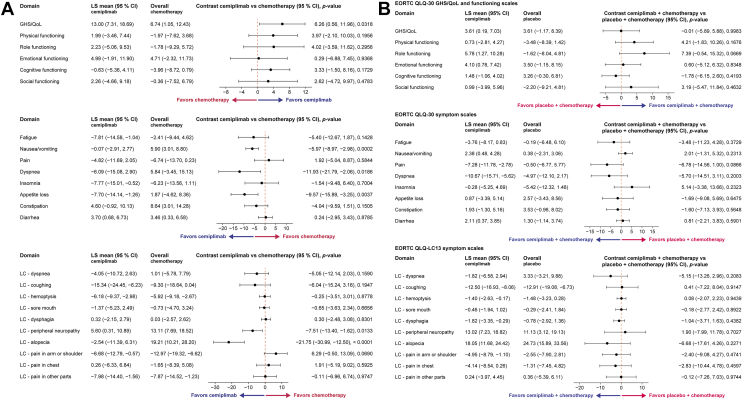
PRO data for the locally advanced NSCLC subgroup across trials. The forest plots reveal estimated differences (treatment versus control) in overall change from baseline (MMRM analysis) in the locally advanced NSCLC subpopulation in (***A***) EMPOWER-Lung 1 and (***B***) EMPOWER-Lung 3 Part 2. For EORTC QLQ-30 GHS/QoL, higher scores indicated that cemiplimab was favored. For EORTC QLQ-30 symptom scales and EORTC QLQ-LC13, lower scores indicated that cemiplimab was favored. LS mean (95% CIs) for the overall hazard ratio in all the patients is demonstrated. No multiplicity adjustments were made. CI, confidence interval; EORTC, European Organisation for Research and Treatment of Cancer; GHS, global health status; LC, lung cancer; LS, least squares; MMRM, mixed-model with repeated measures; PRO, patient-reported outcome; QLQ-LC13, Quality of Life—Lung Cancer 13; QLQ-C30, Quality of Life—Core 30; QoL, quality of life.

The PRO analysis from EMPOWER-Lung 1 revealed that cemiplimab resulted in a significantly favorable overall change from baseline in global health status (GHS)/quality of life (QoL; cemiplimab versus chemotherapy: 6.26; 95% CI: 0.56–11.96). Favorable outcomes for cemiplimab as compared with chemotherapy were also found for symptoms of nausea/vomiting (−5.97; 95% CI: −8.97, −2.98), dyspnea (−11.93; 95% CI: −21.79, −2.06), and appetite loss (−9.57; 95% CI: −15.89, −3.25) per the European Organisation for Research and Treatment of Cancer (EORTC) Quality of Life—Core 30 (QLQ-C30) scale, and symptoms of peripheral neuropathy (−7.51; 95% CI: −13.40, −1.62) and alopecia (−21.75; 95% CI: −30.99, −12.50) per the EORTC Quality of Life—Lung Cancer 13 (QLQ-LC13) scale (Fig. 2*A*).

For EMPOWER-Lung 3, no statistical difference was observed in GHS/QoL between treatment arms in overall change from baseline and across all symptoms and functional scales per the EORTC QLQ-C30 and EORTC QLQ-LC13 (Fig. 2*B*). For both trials, no analyses yielded statistically significant PRO results favoring chemotherapy when comparing between arms for any EORTC QLQ-C30 or EORTC QLQ-LC13 scales (Fig. 2).

### Outcomes for Metastatic NSCLC Subgroups in the EMPOWER-Lung Trials

To provide context for the efficacy outcomes of the locally advanced NSCLC subpopulation, we also assessed efficacy outcomes for the metastatic NSCLC groups in the EMPOWER-Lung trials. Baseline demographic and clinical characteristics are reported in Supplementary Table 1, and OS and PFS outcomes are found in Supplementary Figure 2. In EMPOWER-Lung 1, cemiplimab led to a median OS of 26.0 months versus 12.5 months with chemotherapy (HR: 0.56; 95% CI: 0.44–0.71); median PFS was 7.0 months versus 5.0 months (HR: 0.50; 95% CI: 0.40–0.62). In EMPOWER-Lung 3, cemiplimab plus chemotherapy led to a median OS of 19.3 months versus 12.4 months with chemotherapy (HR: 0.64; 95% CI: 0.49–0.83); median PFS was 6.7 months versus 4.6 months (HR: 0.58; 95% CI: 0.46–0.74). For the treatment and control arms, the ORR was 46.0% versus 19.2% for EMPOWER-Lung 1 and 41.2% versus 20.8% for EMPOWER-Lung 3, respectively (Supplementary Table 2). These efficacy outcomes for the metastatic disease population were largely similar to those observed in the locally advanced NSCLC subgroups, which indicate the absence of significant bias toward more favorable prognosis in patients with locally advanced NSCLC in these trials.

## Discussion

Despite advances in therapeutic options, care for patients with locally advanced NSCLC remains challenging due to complex and heterogenous disease characteristics and the existence of many decision points in diagnosis and treatment. For patients with unresectable locally advanced NSCLC, concurrent CRT followed by durvalumab consolidation has become the standard of care, with a 5-year survival rate of up to 43% in the PACIFIC trial.^6^^,^^7^ In PACIFIC, patients eligible for adjuvant durvalumab must have been able to undergo two or more cycles of concurrent CRT with no evidence of disease progression after CRT, thus limiting the population who can benefit from this regimen.

In clinical practice, many patients with unresectable locally advanced NSCLC are not candidates for concurrent CRT. Large tumor size, poor lung function, prior radiation exposure, tumor infiltration into a large vessel, and risk of hemorrhage can all lead to ineligibility for radiotherapy and therefore preclude patients from receiving curative-intent CRT. Clinicians may also consider patients’ performance status, comorbidities, age, and other individual risk factors.^9^^,^^10^ The proportion of patients with unresectable stage 3 NSCLC who are not candidates for concurrent CRT in real-world clinical practice is unclear. Prevalence can be difficult to estimate due to the heterogenous population, evolving standard of care, and individualized decisions. Limited treatment options are available for this population, and they have been systematically excluded from clinical trials. Although these patients are largely treated as metastatic patients, there remain limited prospective clinical data in this population.^24^

This unmet clinical need was addressed in the phase 3 EMPOWER-Lung trials, where patients with unresectable locally advanced NSCLC who were not candidates for curative-intent CRT were included. Our data demonstrated the clinical benefit of first-line cemiplimab monotherapy in patients with PD-L1 more than or equal to 50% or in combination with chemotherapy independent of PD-L1 expression. The relative improvement versus chemotherapy in patients with locally advanced NSCLC was largely similar to that observed in patients with metastatic disease. These data confirm that clinical course is a suitable alternative in patients with locally advanced NSCLC, who often have poor clinical characteristics making the utilization of curative-intent CRT difficult.

The safety profile of cemiplimab in patients with locally advanced NSCLC was generally consistent with that reported for the overall study populations in EMPOWER-Lung 1 and EMPOWER-Lung 3. The PRO assessments favored cemiplimab monotherapy versus chemotherapy, with improved outcomes in GHS/QoL and in select symptoms (nausea/vomiting, dyspnea, appetite loss, peripheral neuropathy, and alopecia). When cemiplimab was used in combination with chemotherapy, PROs were similar to chemotherapy alone, without significant differences between the treatment groups. These results together further support the benefit–risk profile of first-line cemiplimab in the locally advanced NSCLC patient population.

The limitation of this analysis is the relatively small sample size. Although the cancer stage at screening (metastatic or locally advanced) was one of the prespecified subgroups in the assessment of primary efficacy, the in-depth subgroup analysis (including longer term data on efficacy, safety, and PROs) was not prespecified. However, to our knowledge, this is the first detailed data set from prospective randomized phase 3 studies reporting the outcome of patients with locally advanced NSCLC treated with first-line immunotherapy.

In conclusion, this analysis provides prospective data in this understudied patient population. The data support the clinical benefits of first-line cemiplimab as monotherapy or in combination with platinum-based chemotherapy for the treatment of patients with locally advanced NSCLC who are not candidates for definitive chemoradiation.

## CRediT Authorship Contribution Statement

**Ewa Kalinka**: Conceptualization, Investigation, Writing – review & editing.

**Igor Bondarenko**: Investigation, Writing – review & editing.

**Miranda Gogishvili:** Investigation, Writing – review & editing.

**Tamar Melkadze:** Investigation, Writing – review & editing.

**Ana Baramidze:** Investigation, Writing – review & editing.

**Ahmet Sezer:** Investigation, Writing – review & editing.

**Tamta Makharadze:** Investigation, Writing – review & editing.

**Saadettin Kilickap:** Investigation, Writing – review & editing.

**Mahmut Gümüş:** Investigation, Writing – review & editing.

**Konstantin Penkov:** Investigation, Writing – review & editing.

**Davit Giorgadze:** Investigation, Writing – review & editing.

**Mustafa Özgüroğlu:** Investigation, Writing – review & editing.

**Ruben G.W. Quek:** Conceptualization; Methodology; Formal analysis; Writing – review & editing.

**Debra A.G. McIntyre:** Conceptualization; Methodology; Formal analysis; Writing – review & editing.

**Eric Kim:** Methodology; Formal analysis; Validation; Writing – review & editing.

**Frank Seebach:** Conceptualization, Methodology, Writing – review & editing.

**Jean-Francois Pouliot**: Conceptualization; Methodology; Formal analysis; Writing – review & editing.

## Data Sharing

Qualified researchers can request access to study documents (including the clinical study report, the study protocol with any amendments, a blank case report form, and a statistical analysis plan) that support the methods and findings reported in this manuscript. Individual anonymized participant data will be considered for sharing once the product and indication have been approved by major health authorities (e.g., FDA, European Medicines Agency, Pharmaceuticals and Medical Devices Agency), if there is legal authority to share the data and there is not a reasonable likelihood of participant reidentification. Submit data-sharing requests to https://vivli.org/.

## Disclosure

Dr. Kalinka reports receiving travel and accommodation support from Gilead, Merck Sharp & Dohme, and Regeneron Pharmaceuticals, Inc.; and honoraria from Bristol Myers Squibb, Merck Sharp & Dohme, Gilead, AstraZeneca, Sanofi, Nektar, and GlaxoSmithKline. Dr. Baramidze reports receiving travel support from Regeneron Pharmaceuticals, Inc. Dr. Sezer reports receiving institutional research support from Roche, Merck Sharp & Dohme, Merck Serono, AstraZeneca, Lilly, Novartis, Johnson & Johnson, Regeneron Pharmaceuticals, Inc., and Sanofi. Dr. Gümüş reports receiving honoraria from Roche, Merck Sharp & Dohme, Gen İlaç, and Novartis. Dr. Penkov reports receiving honoraria from AstraZeneca, BeiGene, GlaxoSmithKline, H3 Biomedicine, Janssen, Nektar, Novartis, Pfizer, Regeneron Pharmaceuticals, Inc., and Sanofi. Dr. Özgüroğlu reports receiving honoraria from Novartis, Roche, Janssen, Sanofi, and Astellas; advisory board fees from Janssen, Sanofi, and Astellas; travel support from Bristol Myers Squibb, Janssen, and AstraZeneca; and speaker support from AstraZeneca. Dr. Quek, Dr. McIntyre, Dr. Kim, Dr. Seebach, and Dr. Pouliot are employees and shareholders of Regeneron Pharmaceuticals, Inc. The remaining authors declare no conflict of interest.

## Footnotes

Note: To access the supplementary material accompanying this article, visit the online version of the *JTO Clinical and Research Reports* at www.jtocrr.org and at http://doi.org/10.1016/j.jtocrr.2025.100947.
