# Supplementary material for: First-Line Cemiplimab for Locally Advanced NSCLC: Updated Subgroup Analyses From the EMPOWER-Lung 1 and EMPOWER-Lung 3 Trials
Source: JTO Clin Res Rep. 2025 Dec 22;7(4):100947. doi: 10.1016/j.jtocrr.2025.100947 (PMC12999338; doi:10.1016/j.jtocrr.2025.100947)
Supplement: Supplementary material [file mmc1.docx]

# Supplementary Methods

## Patient-Reported Outcomes

Health-related quality-of-life assessments were measured prior to performing procedures at study visits using the European Organisation for Research and Treatment of Cancer (EORTC) Quality of Life-Core 30 (QLQ-C30) and Quality of Life-Lung Cancer 13 (QLQ-LC13) questionnaires. Patient-reported outcomes (PROs) were assessed at baseline and Day 1 of each treatment cycle for the first six cycles, at Day 1 of every third cycle, and at the end of treatment.

EORTC QLQ-C30 is a 30-item questionnaire comprising a global health status/quality of life subscale, five multiple-item functional subscales, three multiple-item symptom scales, and six single-item symptom scales assessing other cancer-related symptoms. The EORTC QLQ-LC13 questionnaire includes single-item and multi-item subscales assessing lung cancer–associated symptoms and therapy side effects.

Item scores for Likert scales were subsequently transformed to a 0–100 scale using a standard algorithm; high scores on functional domains and low scores on symptoms reflect better outcomes. A change in score of ≥10 points in the transformed score was considered to be clinically meaningful. Mixed-model for repeated measures (MMRM) analyses were performed to compare overall change from baseline scores between the two treatment arms, controlling for baseline.

## Statistical Methods

For EMPOWER-Lung 1, the primary endpoints were analyzed by a stratified log-rank test with the use of tumor histology as stratification factor. Hazard ratios (HRs) and associated 95% confidence intervals (CIs) were estimated by a stratified Cox regression model by means of the treatment as the covariate and tumor histology as the stratification factor. Median overall survival (OS) and progression-free survival (PFS) were estimated by means of the Kaplan–Meier method. For the OS analysis, patients who did not have events (including those who dropped out or were lost to follow-up) were censored at the time of the last contact. For the PFS analysis, patients who did not have events were censored at the time of the last tumor assessment. The objective response rate (ORR) and associated odds ratio were analyzed by means of the Cochran–Mantel–Haenszel test, stratified by tumor histology. The ORR and associated 95% CIs were calculated by the Clopper–Pearson method for each treatment.[^1^](#_ENREF_1)

For EMPOWER-Lung 3 Part 2, OS was analyzed by a stratified log-rank test, using histology and the programmed cell death-ligand 1 (PD-L1) expression level as stratification factors. HRs and 95% CIs were estimated by a stratified Cox regression model, using the treatment as the covariate and adjusted by the same stratification factors. PFS was analyzed using the same statistical method as in the OS analysis. The ORR was analyzed using the Cochran–Mantel–Haenszel test, stratified by histology and PD-L1 expression. Duration of response was analyzed using the Kaplan–Meier method for each treatment arm. The primary endpoint of OS and the secondary endpoints of PFS and ORR were tested hierarchically, in the order of OS, PFS, and ORR.[^1^](#_ENREF_1)

Statistical methods for PROs were as follows. For EMPOWER-Lung 1, the MMRM analysis included only patients with a baseline and >1 post-baseline score. Estimates are based on a MMRM treating time categorical: change from baseline = treatment arm + timepoint + baseline PRO value + histology + geographical region + timepoint × baseline PRO value + timepoint × treatment arm.

For EMPOWER-Lung 3 Part 2, the analysis included only patients with a baseline and >1 post-baseline score, plus all visits up to Cycle 15. Estimates are based on a MMRM treating time categorical: change from baseline = treatment arm + timepoint + baseline PRO value + histology + levels of PD-L1 + timepoint × baseline PRO value + timepoint × treatment arm.

# Supplementary References

1. Sezer A, Kilickap S, Gümüş M, et al. Cemiplimab monotherapy for first-line treatment of advanced non-small-cell lung cancer with PD-L1 of at least 50%: A multicentre, open-label, global, phase 3, randomised, controlled trial. *Lancet*. 2021;397(10274):592-604. doi:10.1016/s0140-6736(21)00228-2

2. Gogishvili M, Melkadze T, Makharadze T, et al. Cemiplimab plus chemotherapy versus chemotherapy alone in non-small cell lung cancer: A randomized, controlled, double-blind phase 3 trial. *Nat Med*. 2022;28:2374-2380. doi:10.1038/s41591-022-01977-y

Supplementary Table 1. Patient Demographics and Baseline Characteristics in Patients With Metastatic NSCLC

| **Characteristic** | **EMPOWER-Lung 1 (n = 565)** | | | **EMPOWER-Lung 3 Part 2 (n = 466)** | | |
| --- | --- | --- | --- | --- | --- | --- |
|  | **Cemiplimab  (n = 239)** | **Chemotherapy  (n = 239)** | **Total  (N = 478)** | **Cemiplimab + chemotherapy (n = 267)** | **Placebo + chemotherapy (n = 130)** | **Total  (N = 397)** |
| Age, years, median (range) | 64 (42‒79) | 64.0 (40‒84) | 64.0 (40‒84) | 63.0 (25‒82) | 63.0 (34‒84) | 63.0 (25‒84) |
| ≥65, n (%) | 110 (46.0) | 114 (47.7) | 224 (46.9) | 113 (42.3) | 50 (38.5) | 163 (41.1) |
| Male, n (%) | 208 (87.0) | 197 (82.4) | 405 (84.7) | 231 (86.5) | 103 (79.2) | 334 (84.1) |
| ECOG PS, n (%) |  |  |  |  |  |  |
| 0 | 61 (25.5) | 63 (26.4) | 124 (25.9) | 41 (15.4) | 15 (11.5) | 56 (14.1) |
| 1 | 178 (74.5) | 176 (73.6) | 354 (74.1) | 224 (83.9) | 113 (86.9) | 337 (84.9) |
| Missing | 0 | 0 | 0 | 2 (0.7) | 2 (1.5) | 4 (1.0) |
| Smoking status, n (%) |  |  |  |  |  |  |
| Current | 84 (35.1) | 76 (31.8) | 160 (33.5) | 145 (54.3) | 62 (47.7) | 207 (52.1) |
| Past | 155 (64.9) | 163 (68.2) | 318 (66.5) | 85 (31.8) | 47 (36.2) | 132 (33.2) |
| Never | 0 | 0 | 0 | 37 (13.9) | 21 (16.2) | 58 (14.6) |
| Histology, n (%) |  |  |  |  |  |  |
| Squamous | 96 (40.2) | 94 (39.3) | 190 (39.7) | 110 (41.2) | 54 (41.5) | 164 (41.3) |
| Non-squamous | 143 (59.8) | 145 (60.7) | 288 (60.3) | 157 (58.8) | 76 (58.5) | 233 (58.7) |
| PD-L1 expression level, n (%) |  |  |  |  |  |  |
| <1% | – | – | – | 80 (30.0) | 37 (28.5) | 117 (29.5) |
| 1–49% | – | – | – | 98 (36.7) | 52 (40.0) | 150 (37.8) |
| ≥50% | 239 (100) | 239 (100) | 478 (100) | 89 (33.3) | 41 (31.5) | 130 (32.7) |

Data cut-off date for EMPOWER-Lung 1: March 4, 2022. Data cut-off date for EMPOWER-Lung 3: June 14, 2022.

ECOG PS, Eastern Cooperative Oncology Group performance status; NSCLC, non-small cell lung cancer; PD-L1, programmed cell death-ligand 1.

Supplementary Table 2. Summary of Tumor response in Patients With Metastatic NSCLC

|  | **EMPOWER-Lung 1^a^ (n = 565)** | | **EMPOWER-Lung 3 Part 2 (n = 466)** | |
| --- | --- | --- | --- | --- |
|  | **Cemiplimab  (n = 239)** | **Chemotherapy  (n = 239)** | **Cemiplimab + chemotherapy (n = 267)** | **Placebo + chemotherapy  (n = 130)** |
| Duration of follow-up, months, median (range)^b^ | 34.76 (24.0–56.0) | 34.79 (24.0–52.8) | 28.29 (20.5–35.9) | 28.25 (21.6–35.8) |
| ORR, n (%) | 110 (46.0) | 46 (19.2) | 110 (41.2) | 27 (20.8) |
| 95% CI | 39.6–52.6 | 14.4–24.8 | 35.2–47.4 | 14.2–28.8 |
| Odds ratio (95% CI) | 3.56 (2.36–5.36) | | 2.67 (1.64–4.36) | |
| Best overall response, n (%) |  |  |  |  |
| Complete response | 21 (8.8) | 5 (2.1) | 9 (3.4) | 0 |
| Partial response | 89 (37.2) | 41 (17.2) | 4 (1.5) | 1 (0.8) |
| Stable disease | 55 (23.0) | 121 (50.6) | 28 (10.5) | 19 (14.6) |
| Non-complete response/non-progressive disease | 2 (0.8) | 1 (0.4) | 101 (37.8) | 27 (20.8) |
| Progressive disease | 50 (20.9) | 44 (18.4) | 22 (8.2) | 24 (18.5) |
| Not evaluable | 22 (9.2) | 27 (11.3) | 103 (38.6) | 59 (45.4) |
| Kaplan–Meier estimated DOR, months, median (95% CI) | n = 110 25.5 (17.1–35.2) | n = 46 5.9 (4.3–7.1) | n = 110 16.0 (12.5–18.7) | n = 27 8.3 (4.3–16.8) |

Radiographic tumor assessments and efficacy endpoints including OS, PFS, ORR, and DOR were previously reported for EMPOWER-Lung 1[^1^](#_ENREF_1) and EMPOWER-Lung 3 Part 2.[^2^](#_ENREF_2)

^a^PD-L1 ≥50% population.

^b^From randomization to data cutoff date (March 4, 2022, for EMPOWER-Lung 1; June 14, 2022, for EMPOWER-Lung 3).

CI, confidence interval; DOR, duration of response; NSCLC, non-small cell lung cancer; ORR, objective response rate; OS, overall survival; PD-L1, programmed cell death-ligand 1; PFS, progression-free survival.

Supplementary Figure 1. Study designs of EMPOWER-Lung 1 and EMPOWER-Lung 3 Part 2.


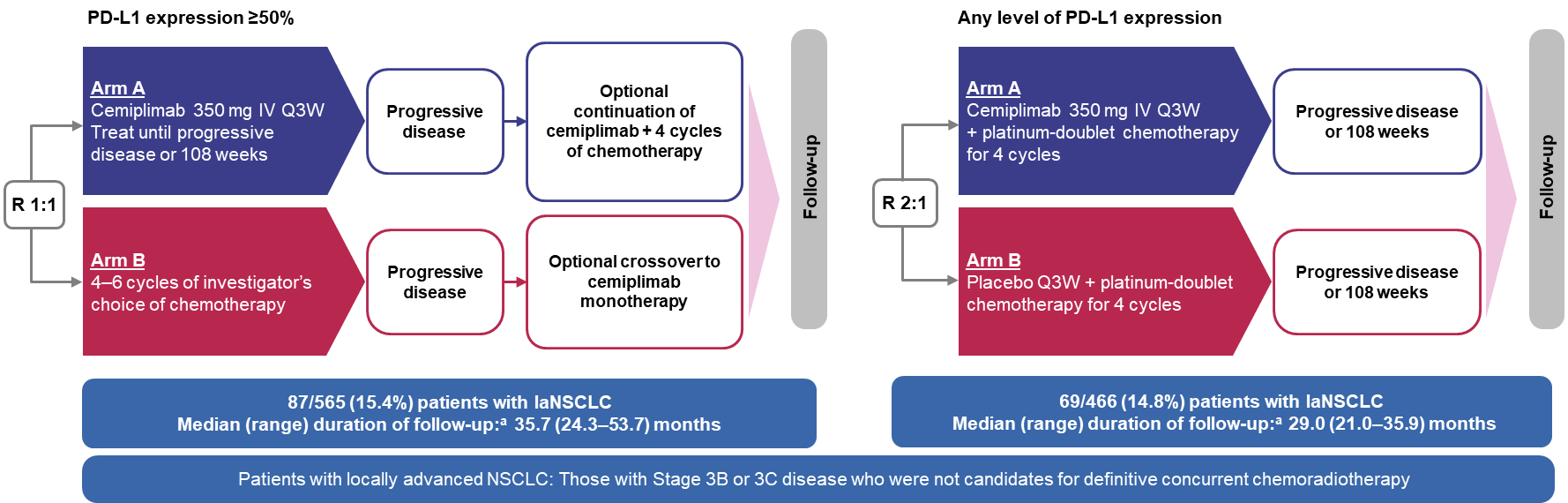


Study designs were previously reported for EMPOWER-Lung 1[^1^](#_ENREF_1) and EMPOWER-Lung 3 Part 2.[^2^](#_ENREF_2)

^a^From randomization to the data cutoff date.

IV, intravenous; laNSCLC, locally advanced non-small cell lung cancer; PD-L1, programmed cell death-ligand 1; Q3W, every 3 weeks; R, randomization.

Supplementary Figure 2. (*A*) OS and (*B*) PFS in patients with metastatic NSCLC.

**A**


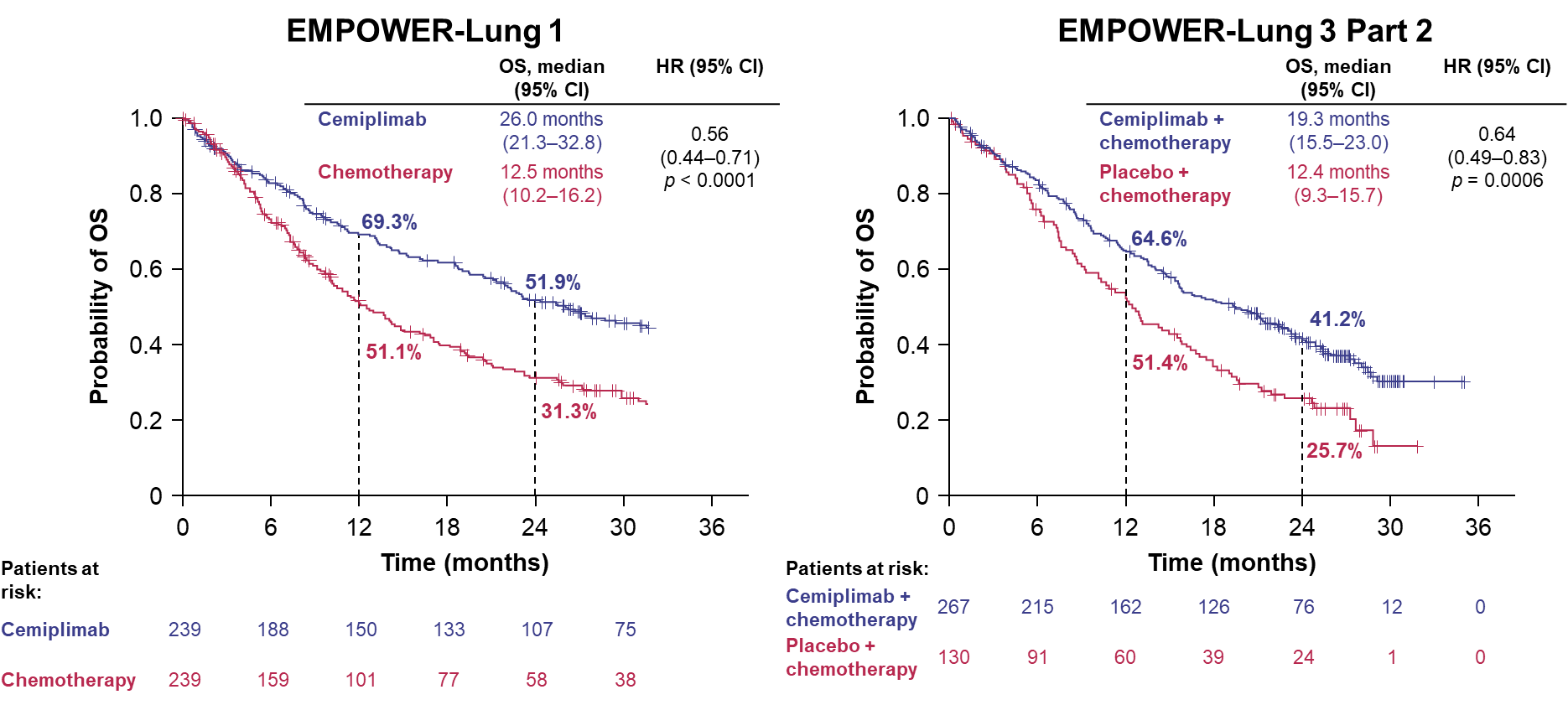


**B**

**
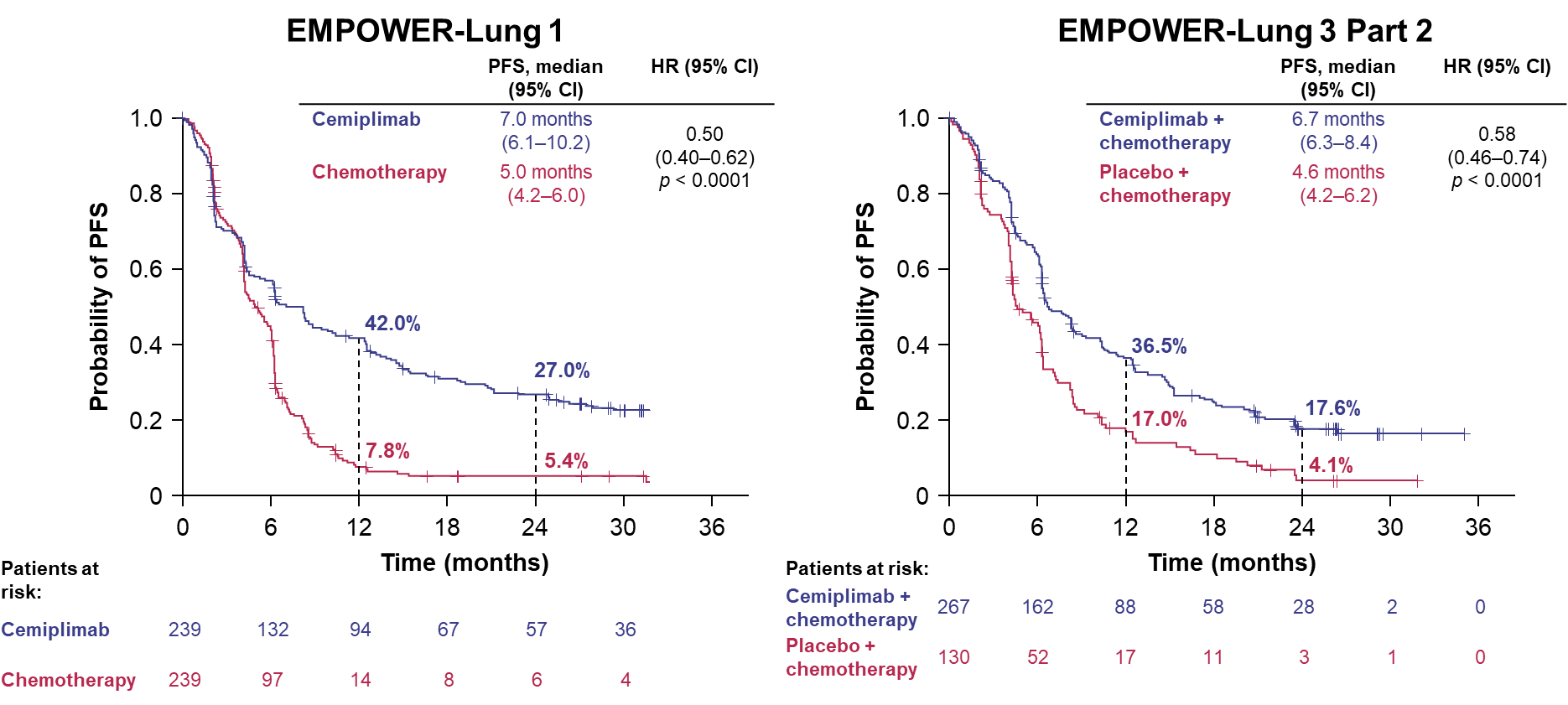
**

Kaplan–Meier curves for OS and PFS were represented for cemiplimab versus placebo and cemiplimab + chemotherapy versus placebo + chemotherapy. Tick marks indicate censored observations, vertical lines indicate the times of landmark OS or PFS analyses, and percentages at the times of landmark PFS analysis indicate the probability of PFS or OS.

CI, confidence interval; HR, hazard ratio; NSCLC, non-small cell lung cancer; OS, overall survival; PFS, progression-free survival.
